# Starching again: manipulating starch metabolism to improve cassava quality

**DOI:** 10.1093/plphys/kiag350

**Published:** 2026-06-10

**Authors:** Rose McNelly

**Affiliations:** Assistant Features Editor, Plant Physiology, American Society of Plant Biologists, Rockville, MD, United States; John Innes Centre, Norwich Research Park, Norwich NR4 7UH, United Kingdom

Cassava (*Manihot esculenta* Crantz) is the world's sixth most important crop in terms of global annual production (Burns et al 2010). It grows well in drought-stricken, nutrient-poor areas and is a starch-rich staple for over a billion people in sub-Saharan Africa, Asia, and Latin America (Adebayo 2023; Borku et al 2025). Beyond food, cassava is used to produce bioplastics, packaging, and paper.

Cassava root yields are determined by starch content. Tracer experiments using ^14^CO_2_ established that cassava roots are a carbon sink during vegetative growth (Hume 1975), and carbon delivered to roots is metabolized into starch. Farmers sometimes prune the shoots off of the root system to feed animals, prevent damage from weather, and prevent postharvest deterioration of the roots (Wheatley and Schwabe 1985). After shoot pruning, roots can become a source of carbon, and starch is remobilized to sustain respiration and canopy regrowth. Starch remobilization limits yield from the roots, so understanding how the sink-to-source transition occurs is of much interest.

In a recent study in *Plant Physiology*, David et al (2026) measured storage root starch content of freshly pruned plants and plants 10 days postpruning that had shoot regrowth. Unsurprisingly, starch content had decreased by 47% 10 days after pruning. There were no significant changes in the amount of simple sugars, like glucose and fructose, which suggests that starch degradation is enabling respiration to maintain sugar levels and promote shoot regrowth. Further experiments to track carbon as it is remobilized from the storage root could determine precisely where the remobilized carbon is utilized.

To explore the sink-to-source transition in more detail, storage root samples were collected along a time course of 4 hours postpruning to 10 days postpruning, and RNA was extracted and sequenced. The greatest transcriptional changes occurred from 2 days postpruning. Notably, there was a reduction in expression of genes involved in respiration, suggesting that respiration, and potentially growth, is restricted in storage roots postpruning. To confirm if growth is reduced, storage root size and mass could be tracked over a time course pre- and postpruning.

From this point onward, the authors focused on starch metabolism. Analysis of their transcriptomic data revealed that from 2 days postpruning, there was downregulation of starch synthesis genes and upregulation of starch degradation genes. The authors highlight 3 highly upregulated genes—*AMY3* and *BAM9*, which participate in starch degradation in Arabidopsis (Seung et al 2013; David et al 2022), and *KINγ*, which regulates carbon allocation (Jamsheer et al 2021). To investigate whether differences in gene expression resulted in changes in protein abundance, shotgun proteomics was conducted with samples collected prior to pruning and 10 days postpruning. The AMY3, BAM9, and KINγ peptides all had significantly greater abundance at the later time point.

Higher abundance of starch-degrading proteins does not necessarily mean that overall enzymatic activity is greater, so to measure enzyme activity zymograms were used. Zymograms are native protein gels in which substrates are incorporated into the gel matrix, allowing assessment of enzymatic activity of crude protein extracts against the incorporated substrates. Band intensities were greater in extracts collected 10 days postpruning than 0 days postpruning on multiple glucan-containing substrates. Correlating bands on the zymogram to specific enzymatic activity is not possible. However, the authors propose that 1 band, which appears more strongly 10 days postpruning, may contain AMY3 based on similarities to the migration pattern of Arabidopsis AMY3 (Yu et al 2005). Combined with the transcriptomics and proteomics, these complementary techniques provide robust evidence that starch degradation is upregulated upon pruning.

The results thus far suggest that *AMY3* may be an important player in storage root starch degradation, so the authors investigated *AMY3* further. Phylogenetic analysis revealed that there are 2 *AMY3*s in cassava—*AMY3a* and *AMY3b*. Comprehensive analysis with reverse transcription quantitative PCR and immunoblotting showed that *AMY3a* is the isoform expressed in storage roots, so all subsequent experiments were performed with *AMY3a*.

Based on the aforementioned results, the authors hypothesized that suppression of *AMY3* in storage roots would limit starch degradation postpruning and enhance cassava yields. Therefore, 6 transgenic cassava lines containing an RNAi construct against *AMY3a* were generated. Four of these had reduced *AMY3a* expression and AMY3a protein abundance that was robust in glasshouse and field conditions. The RNA interference (RNAi) plants were grown in a field in Nigeria and pruned, and *AMY3a* expression and storage root starch content were measured at 0 days and 10 days postpruning. The increase in *AMY3a* expression postpruning was significantly suppressed in RNAi lines. The suppression of *AMY3a* expression was associated with a reduction in starch remobilization—starch content of wild-type plants decreased by 30% 10 days postpruning, yet in RNAi lines, the reduction was only 4% to 17%. To test whether the reduction in starch remobilization affected plant physiology, agronomic properties were measured. No differences were observed in resprouting capacity or the fresh weight of storage roots produced, but there was a reduction in the harvest index of the transgenic lines. The reduction in harvest index is likely to be a result of somaclonal variation introduced during tissue culture, as all the plants that had gone through transgenesis and tissue culture, even those without *AMY3a* suppression, had a reduction in harvest index. Together, these results suggest that *AMY3a* suppression improves cassava storage root yields without having a detrimental effect on shoot regrowth. In the future, it may be interesting to generate *amy3a* full knockout mutants. Would these plants have even less starch remobilization and greater yields? Or would complete elimination of *AMY3a* inhibit shoot regrowth and cause unwanted side effects?

In summary, David et al (2026) identified that in cassava, *AMY3a* is upregulated postpruning for starch remobilization to support regrowth (Fig. 1a,b). Promisingly, the suppression of *AMY3a* (Fig. 1c) reduced starch remobilization but had no effect on shoot regrowth and could be a method for increasing starch content in cassava storage roots and improving yields and quality. Beyond identifying *AMY3a*, this study lays the foundations for future work. The comprehensive transcriptomics and proteomics reveal other candidates involved in postpruning starch degradation. It would be interesting to suppress the other candidates, either individually or in combination with *AMY3a*, and measure the effect on yield and shoot regrowth. What is still unclear is whether there is a minimum amount of starch remobilization sufficient for shoot regrowth. Discovering whether a minimum amount exists could allow us to engineer a cassava plant with precisely this amount of starch remobilization, therefore maximizing yields and quality without negatively affecting plant regrowth.

**Recent related articles in *Plant Physiology*:**

- Rüscher et al (2024) investigated source-sink dynamics in cassava and revealed that phloem transport is a major contributor to carbon allocation.
- Heutinck et al (2025) described how *AMY3* is involved in starch granule initiation in Arabidopsis mutants lacking 2 debranching enzymes.

**Figure 1:**
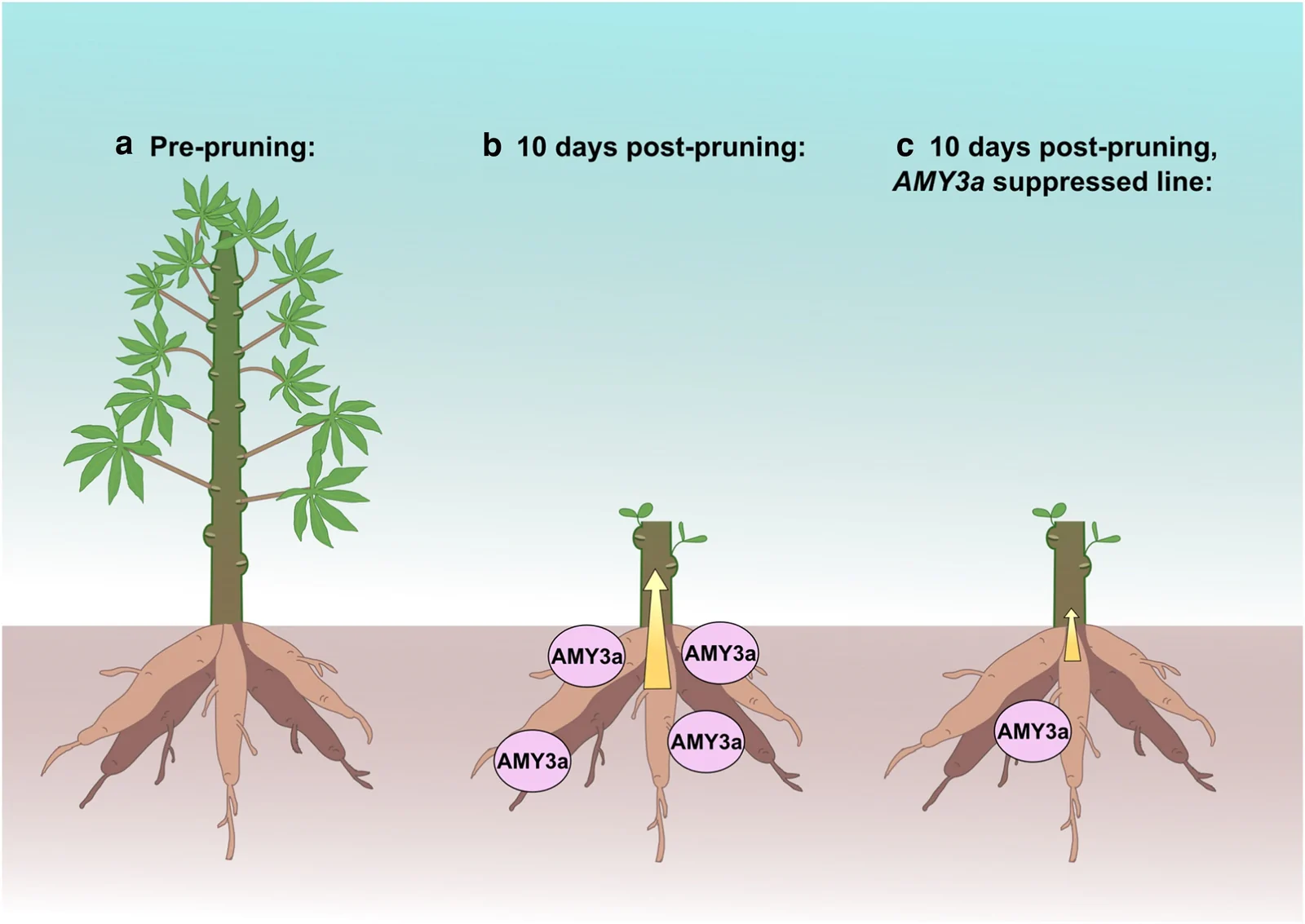
AMY3a is involved in starch remobilization postpruning in cassava. a) Cassava plant prepruning. b) A cassava plant approximately 10 days postpruning. There is increased abundance of the AMY3a protein in the storage roots that facilitates starch remobilization (yellow arrow) from the roots to support shoot regrowth. c) An *amy3a*-suppressed cassava plant approximately 10 days postpruning. Reduced abundance of AMY3a leads to reduced starch remobilization without influencing shoot regrowth. The amount of starch remobilization is represented by the size of the yellow arrow. This figure was prepared using Inkscape.

## Data Availability

No new data were generated or analyzed in support of this research.
